# Supplementary material for: Evaluation of Serum Biomarkers and Electroencephalogram to Determine Survival Outcomes in Pediatric Post-Cardiac-Arrest Patients
Source: Children (Basel). 2023 Jan 18;10(2):180. doi: 10.3390/children10020180 (PMC9955226; doi:10.3390/children10020180)
Supplement: Supplementary file 1 [file children-10-00180-s001.zip › children-2046341-supplementary.pdf]

Laboratory investigations

1) Complete blood count (CBC) with differential:
Two mL of fresh venous blood was collected for CBC in a tube containing ethylenediaminetetraacetic acid as an anticoagulant. CBC was performed using Sysmex XT-1800i (Sysmex, Kobe, Japan).

2) C-reactive protein (CRP) was measured using the semi-quantitative latex agglutination test (Avitex CRPkit, Omega Dignostic Li mited, Scotland, United Kingdom).

3) Other laboratory investigations:
a. Serum 100 B assessment
b. Neuron-specific enolase assessment

Specimen (serum):

Blood samples for Neuron-specific enolase and S100B were withdrawn into plastic syringes and quickly transferred to chilled tubes. Serum was separated by means of immediate refrigerated centrifugation (3000 rpm) at 4ºC for 20 minutes. The serum samples obtained were kept frozen below -20 °C until assay.

a. Serum 100B assessment:

Serum S100B levels assessment was done using enzyme-linked immune-sorbent assay (ELISA) kit (Human S100 Calcium Binding Protein B (S100B) ELISA KIT catalogue number 201-12-4851).

Principle of the Test
The kit uses a double-antibody sandwich enzyme-linked immunosorbent assay (ELISA) to assay the level of Human S100 Calcium Binding Protein B (S100B) in samples.
A monoclonal antibody specific for S100B has been pre-coated onto a microplate. Standards and samples are pipetted into the wells and any S100B present is bound by the immobilized antibody. After washing away any unbound substances, an enzyme-linked polyclonal antibody specific for S100B is added to the wells. Following a wash to remove any unbound antibody-enzyme reagent, a substrate solution is added to the wells and color develops in proportion to the amount of S100B bound in the initial step. When the color development completes, the intensity of the color is measured.

Assay procedure:
1. Standard dilution:
120 ul original standard +120 ul Standard dilutents Standard no. 5 2000ng/L
120 ul Standard no. 5 +120 ul Standard dilutents Standard no. 4 1000ng/L
120 ul Standard no. 4 +120 ul Standard dilutents Standard no. 3 500ng/L

120 ul Standard no. 3 +120 ul Standard dilutents Standard no. 2 250ng/L
120 ul Standard no. 2 +120 ul Standard dilutents Standard no. 1 125ng/L

2. Blank well: Don't add samples and S100B-antibody labeled with Biotin, Streptavidin-HRP. Only chromogen solution A and B and stop solution are allowed.
3. Standard wells: Add standard 50 uL, streptavidin –HRP 50 uL (the standard has already combined biotin antibody, it is not necessary to add the antibody).

4. To be tested wells: Add sample 40 uL and then add both S100B –antibody 10 uL and streptavidin –HRP 50 uL. Then, seal the sealing membrane, gently shake, and incubate for 60 minutes at 37ºC.

5. Washing: Remove the membrane carefully, drain the liquid, and shake away the remaining water.

6. Add chromogen solution A 50 ul, then chromogen solution B 50 uL to each well. Gently mix and incubate for 10 minutes at 37ºC away from light.

7. Stop: Add Stop Solution 50 uL into each well to stop the reaction (the blue changes into yellow immediately)

8. Final measurement: Take blank well as zero, measure the optical density (OD) under 450 nm wavelength which should be carried out within 15 minutes after adding the stop solution.

9. According to standards' concentration and the corresponding OD values, calculate out the standard curve linear regression equation, and then apply the OD values of the sample on the regression equation to calculate the corresponding sample's concentration.

Calculation:
Take the standard density as the horizontal, the OD value for the vertical, draw the standard curve on graph paper, find out the corresponding density according to the sample OD value by the sample curve (the result is the sample density) or calculate the straight line regression equation of the standard curve with the standard density and the OD value, with the sample OD value in the equation, and calculate the sample density.

b. Neuron-specific enolase assessment:

Serum Neuron-specific enolase levels assessment was done using Cobas e411 (ROCHE diagnostic) fully automated system. The NSE in the sample reacts with a biotinylated monoclonal NSE-specific antibody and a ruthenium labelled NSE –specific monoclonal antibody. After addition of streptavidin –coupled micro particles the antigen/antibody complex is detected using chemiluminescence. The result is determined via an instrument specific calibration curve and presented in ng/L.
